# Evaluation of the efficiency of dried blood spot-based measurement of hepatitis B and hepatitis C virus seromarkers

**DOI:** 10.1038/s41598-020-60703-1

**Published:** 2020-03-02

**Authors:** Chikako Yamamoto, Shintaro Nagashima, Mitsuo Isomura, Ko Ko, Channarena Chuon, Tomoyuki Akita, Keiko Katayama, Joseph Woodring, Md. Shafiqul Hossain, Kazuaki Takahashi, Junko Tanaka

**Affiliations:** 1 Department of Epidemiology, Infectious Disease Control and Prevention, Graduate School of Biomedical and Health Science, Hiroshima University, Hiroshima, Japan; 2 Department of Clinical Evaluation, Fujirebio Inc., Tokyo, Japan; 3 Expanded Program on Immunization Unit, Division of Communicable Diseases, World Health Organization Regional Office for the Western Pacific Country Office, Manila, Philippines; 4 Expanded Program on Immunization, World Health Organization Country Office, Phnom Penh, Cambodia; 5 Present Address: Behavioral and Clinical Research Section, HIV/STD Research Program, Thailand MOPH - U.S. CDC Collaboration (TUC), Nonthaburi, Thailand

**Keywords:** Hepatitis B, Hepatitis C

## Abstract

Although hepatitis B (HBV) and C (HCV) virus infections are still global health issues, measuring sero-markers by standard venipuncture is challenging in areas limited with the adequate human resources and basic infrastructure. This study aimed to inform the usefulness of dried blood spot (DBS) sampling technique for epidemiological study of HBV and HCV in the resources limited areas. We compared specimen recovery rate expressed as analytical sensitivity ratio of HBsAg, HBcAb and anti-HCV between serum specimens and DBS samples (HemaSpot vs Whatman903). Sensitivity ratio was calculated as the ratio of the measured value from DBS to the measured value from serum. Then both the qualitative and quantitative comparisons of HBsAg detection by DBS were done using Cambodian samples. HBsAg, HBcAb and anti-HCV sensitivity ratios for the highest sample dilution (8-fold) were 31.2:1, 38.9:1 and 32.0:1 for Whatman903 card and 17.6:1, 23.5:1 and 26.3:1 for HemaSpot respectively. Detection efficacy of HemaSpot (80%) was not inferior to Whatman903 (60%) after 1 month storage, and no significant difference in any hepatitis virus sero-markers was observed in HemaSpot-spotted patient samples stored for 2 weeks at −25 °C and 29 °C. All reference HemaSpot -spotted 400 HBsAg sero-negative samples showed negative. Sensitivity and specificity of HBsAg in HemaSpot were 92.3% and 100%. The recovery expressed as analytical sensitivity ratio of HBsAg, HBcAb and anti-HCV of HemaSpot specimen were not inferior to Whatman903*.* Therefore, DBS with its usefulness proved as an acceptable tool for large epidemiological study of HBV and HCV in resources limited remote area.

## Introduction

Hepatitis B virus (HBV) and hepatitis C virus (HCV) constitute a global health issue. Although there are the effective hepatitis B vaccine since 1982^1^ and anti-viral drugs such as interferon alpha, lamivudine and others (tenofovir, adefovir, etc)^2,3^, WHO reported that approximately 257 million people have been infected with HBV and only 16.7% of the people diagnosed with hepatitis B were on anti-viral treatment as of 2016^4^. Similarly, even there is no vaccine for HCV till now, the direct-acting antiviral drugs (DAAs) for the treatment of HCV were initially developed in 2011^5^ and have since yielded high sustained virologic responses (SVRs)^6^. But, WHO reported that 71 million people have been chronically infected with HCV and approximately 19% of global population (13.1 millions) knew their diagnosis and around 5 million only had treated with DAA at the end of 2017^7^. Therefore, the World Health Organization (WHO) announced in 2016 a target to eliminate HBV as public health threats by 2030 by reducing the hepatitis B surface antigen (HBsAg) prevalence among children to ≤0.1%^8^ and to eliminate HCV by 90% reduction in new diagnosis and 65% reduction in HCV related mortality by 2030^9^. However, both the expected or unexpected barriers are still existed particularly in remote areas for its less accessibility, high cost and require human and technical resources to eliminate HBV and HCV in the world^10,11^.

Understanding the particular disease burden *in situ* in particular countries is the essential key to meet the WHO 2030 elimination goal of either HBV or HCV. The disease burden may vary geographically in different parts of the world^12,13^. Given the widespread nature of HBV and HCV, global epidemiological surveys are needed to fully understand the infection rates and prevalence. To investigate the ground situation of the infections, the screening and the surveillance are the core principles. Although the sampling by gold standard venipuncture requires well-experienced human resources, equipment and infrastructure, it is difficult to conduct epidemiological surveys of factors such as viral seromarkers in resources constraint settings with limited adequate human resources and basic infrastructures^14^. So that it makes barriers in the prevention, control and its clinical management.

The dried blood spot (DBS) is a non-invasive relatively new blood sampling technology that requires the collection of a very small amount of blood. Specifically, a blood sample is obtained from a finger prick and dropped onto a filter paper, dried and stored for subsequent analysis. Accordingly, this may be a more practical option for resource-limited areas. However, very few published reports have discussed DBS, and most have used the Whatman903 card^14–25^.

Although HemaSpot-HF blood collecting device (Spot on Science Inc., Austin, USA) is a newly developed DBS in recent years, it represents an affordable alternative to serum-based evaluation of viral hepatitis infections. However, there is no report assessing the feasibility of hepatitis virus marker measurements using HemaSpot, and there are very few reports on its effectiveness^14–17,26^. So far, no report has directly compared the sensitivity of the Whatman903 card with the HemaSpot (another type of DBS) and standard serum analysis^14–17,26^ for measuring hepatitis virus seromarkers. As the amount of blood on the punched filter paper of Whatman903 depends on the initial spread of blood on the filter paper and the position of punching, it is difficult to accurately assess the blood volume on the paper and, therefore, the extraction efficiency. Thus, the evaluation of the sensitivity ratios of various blood collection methods that use absorbent/filter papers, rather than validation of the extraction efficiency, should be considered the conventional method. In this context, sensitivity can be estimated as the ratio of the measured value from DBS samples to the measured value from serum samples^14^. Therefore, this study aimed to evaluate the usefulness of DBS sampling technique for epidemiological study of HBV and HCV in the resources limited areas by comparing the specimen recovery rate expressed as analytical sensitivity ratio of HBsAg, HBcAb and anti-HCV between serum specimen and DBS samples.

## Results

### Comparison of HBsAg sensitivity ratios from serum samples, Whatman903 and HemaSpot

**Figure 1:**
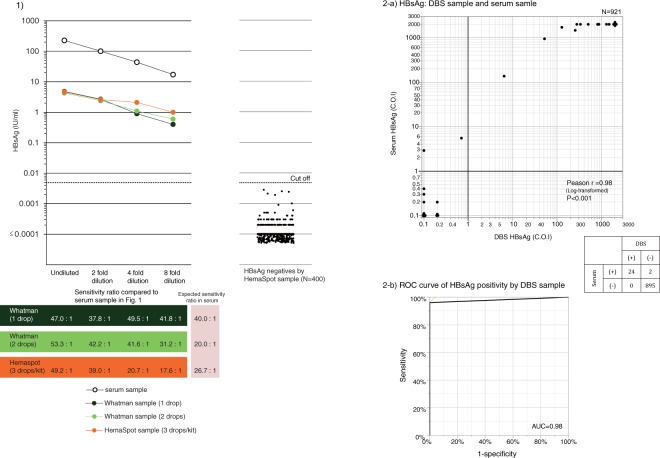
Hepatitis B virus surface antigen (HBsAg) sensitivity ratios for Whatman903 and HemaSpot samples relative to serum samples. **(a)** The sensitivity ratio of each DBS sample is presented relative to the direct serum sample (gold standard). White, orange, dark green and light green dots represent the sensitivity ratios of serum, HemaSpot and Whatman903 at one drop/circle and two drops/circle, respectively. Each spot indicates the resultant ratios obtained at different dilutions (undiluted, 2-fold, 4-fold, 8-fold). The dot plot represents the measured HBsAg values from HBsAg-seronegative HemaSpot samples from a general Cambodian population (n = 400). (**b**) (i) The correlation between the serum measurement value and the DBS sample measurement value of the general population in Cambodia (n = 921) is shown in the scatter plot. Black solid line was drawn at the cutoff value of HBsAg. The breakdown of HBsAg positive numbers and negative numbers of serum specimens and DBS specimens is shown in the table. (ii)The vertical axis shows the sensitivity ratio, the horizontal axis shows the false positive rate (1 - specificity). ROC curve was represented based on the measurement result of DBS specimen.

For the artificially prepared HBsAg samples in whole blood, direct serum measurement yielded the quantitative titer of HBsAg in the range of 17.2–227.4 IU/ml for undiluted to 8-fold diluted solutions. As for Whatman903 at one drop/circle, two drops/circle and HemaSpot, the quantitative titers of HBsAg were measured in the range of 0.4–4.8, 0.6–4.3 and 1.0–4.6 IU/ml, respectively. Therefore, the estimated sensitivity ratios were 40.0 (41.8–47.0) for Whatman903 at one drop/circle, 20.0 (31.2–53.3) for Whatman903 at two drops/circle and 26.7 (17.6–49.2) for HemaSpot three drops/ kit (Fig. 1a).

### Specificity of HBsAg titer measured using HemaSpot

All HBsAg-seronegative HemaSpot samples (n = 400; confirmed by Rapid-POC and whose specimens were collected using DBS only) obtained during the nationally representative survey in Cambodia yielded HBsAg quantitative titers of ≤0.005 IU/ml (Fig. 1a) showing the negative to HBsAg.

### Rate of agreement, sensitivity and specificity of HBsAg in HemaSpot

The rate of agreement of HBsAg qualitative measurement between Rapid test and HemaSpot-spotted samples from 921 Cambodian residents using kappa coefficient is 0.978 (95%CI: 0.94–1.0%, Supplementary Table S1a). Moreover, HBsAg from HemaSpot samples and serum specimens were measured quantitatively for 921 Cambodian residents (whose specimens were collected using both venipuncture and DBS) and shown in Supplementary Table S1b. HBsAg prevalence of serum sample was 2.8% (26/921) and HBsAg prevalence of DBS samples was 2.6% (24/921) (Fig. 1b-i). AUC of HemaSpot was 0.98, with sensitivity of 92.3% and specificity of 100% (Fig. 1b-ii).

### Comparison of HBcAb and anti-HCV sensitivity ratios between Whatman903 and HemaSpot samples

**Figure 2:**
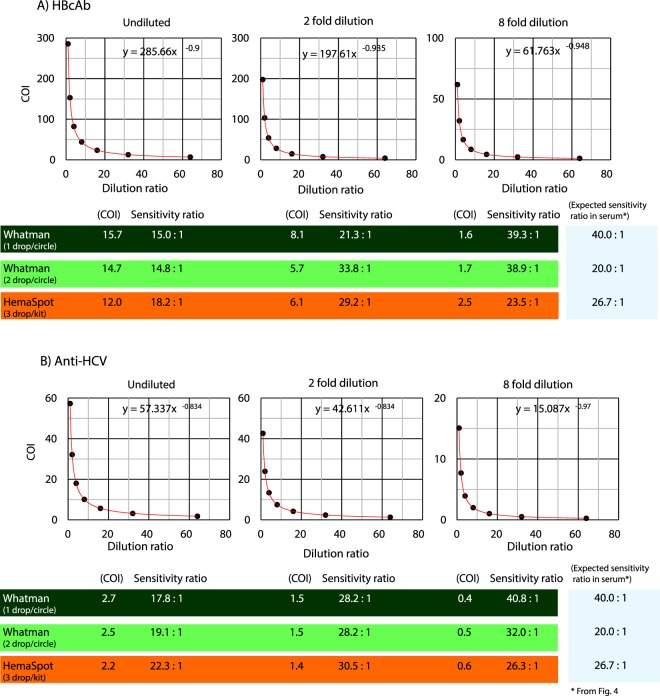
Hepatitis B virus core antibody (HBcAb) and anti-hepatitis C virus (anti-HCV) calibration curves and sensitivity ratios for Whatman903 and HemaSpot samples relative to serum samples. The graphs depict the calibration curves and sensitivity ratios for (**A**) HBcAb and (**B**) Anti-HCV and plot the dilution ratio (x-axis) versus the COI (y-axis) for different dilutions (undiluted, 2-fold, 8-fold) and different types of DBS (Whatman903 at one drop/circle and two drops/circle and HemaSpot).

The HBcAb sensitivity ratios for the two different types of DBS were estimated using the calibration curves (solutions ranging from undiluted to 8-fold dilution). The sensitivity ratios were in the range of 15.0–39.3, 14.8–38.9 and 18.2–23.5 for Whatman903 at one drop/circle and two drops/circle and HemaSpot, respectively (Fig. 2A, Table 1). Similarly, the anti-HCV sensitivity ratios were also calculated from the calibration curves, which yielded values in the range of 17.8–40.8, 19.1–32.0 and 22.3–26.3 in Whatman903 at one drop/circle and two drops/circle and HemaSpot respectively (Fig. 2B, Table 1).

**Table 1:** Measurement result of hepatitis B virus core antibody (HBcAb) and anti-hepatitis C virus (anti-HCV) in Whatman903 and HemaSpot samples.

Marker	DBS type and number of drop	Dilution ratio	1^st^ measure	2^nd^ measure	Mean	SD
HBcAb	Whatman 1drop	x1	20.4	11.0	15.7	6.6
		x2	8.6	7.5	8.1	0.8
		x8	1.7	1.4	1.6	0.2
	Whatman 2drop	x1	12.7	16.6	14.7	2.8
		x2	7.2	4.1	5.7	2.2
		x8	2.2	1.2	1.7	0.7
	Hemaspot 3drop	x1	13.1	10.9	12.0	1.6
		x2	5.4	6.7	6.1	0.9
		x8	2.6	2.4	2.5	0.1
HCV Ab	Whatman 1drop	x1	2.7	2.6	2.7	0.1
		x2	1.5	1.4	1.5	0.1
		x8	0.4	0.4	0.4	0.0
	Whatman 2drop	x1	2.5	2.5	2.5	0.0
		x2	1.5	1.4	1.5	0.1
		x8	0.5	0.5	0.5	0.0
	Hemaspot 3drop	x1	2.2	2.2	2.2	0.0
		x2	1.3	1.4	1.4	0.1
		x8	0.6	0.6	0.6	0.0

The table shows the duplicate measurement values and SD of HBcAb and anti-HCV in different types of DBS (Whatman903 at one drop/circle and two drops/circle and HemaSpot).

### Stability of HBsAg, HBcAb and anti-HCV in Whatman903 and HemaSpot-spotted artificial samples after 1 month at different storage temperatures

**Figure 3:**
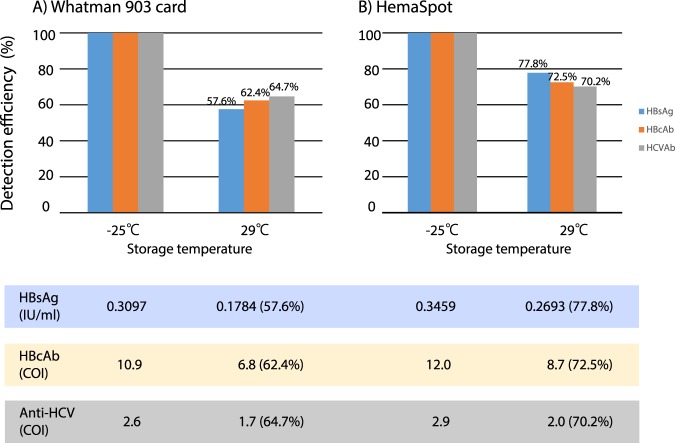
The detection efficacy of hepatitis B virus (HBV) soluble antigen (HBsAg), HBV core antibody (HBcAb) and anti-hepatitis C virus (anti-HCV) in Whatman903 and HemaSpot samples stored for 1 month at different storage temperatures. Each graph indicates the HBsAg, HBcAb and Anti-HCV detection efficiencies (blue, orange and light brown bars, respectively) in (**A**) Whatman903 and (**B**) HemaSpot samples. All samples were tested following a 1-month exposure to different storage temperatures (−25 °C or 29 °C).

At 1 month after storage at room temperature (29 °C), the detection efficiencies of HBsAg, HBcAb and anti-HCV relative to those obtained from samples stored at −25 °C were 57.6–64.7% for Whatman903 samples and 70.2–77.8% for HemaSpot samples (Fig. 3).

### Stability of HBsAg, HBcAb and anti-HCV in HemaSpot-spotted patients samples after 2 weeks at different storage temperatures

**Figure 4:**
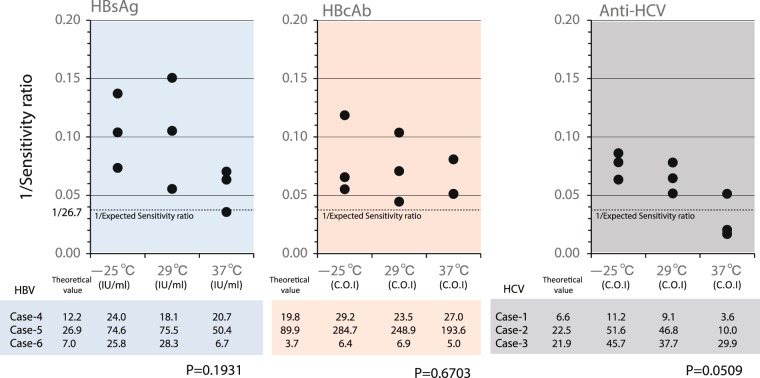
The sensitivity ratios of HBsAg, HBcAb and Anti-HCV in HemaSpot samples stored for 2 weeks at different temperatures (relative to serum samples). Each graph represents the sensitivity ratio of HBsAg (hepatitis B virus surface antigen; first graph), HBcAb (hepatitis B virus core antibody; second graph) or anti-HCV (hepatitis C virus antibody; third graph) in samples that had been stored at different temperatures (−25 °C, 29 °C or 37 °C) for 2 weeks. Cases 1, 2 and 3 are samples collected from HCV-infected patients; cases 4, 5 and 6 are samples from HBV-infected patients.

Compared to patient serum samples (HBV cases 4–6) stored at −25 °C for 14 days, the sensitivity ratios of HBsAg for HemaSpot samples stored at −25 °C, 29 °C and 37 °C. In case 4, it was 18.1–24.0 against the theoretical value of 12.22, in case 5 it was 50.4–75.5 against the theoretical value of 26.88, and in case 6 it was 6.7–28.3 against the theoretical value of 7.04 (Fig. 4). Compared to patient serum samples (HCV cases 1–3) stored at −25 °C for 14days, it was 3.6–11.2 against the theoretical value of 6.62 in case 1, 10.0–51.6 against the theoretical value of 22.47 in case 2, and 29.9–45.7 against the theoretical value of 21.87 in case 3 (Fig. 4).

## Discussion

As noted in introduction and previous reports, DBS methods such as the Whatman903 card are considered useful for measurement of hepatitis virus seromarkers in resource-limited areas^16,20^. By the qualitative comparison of HBsAg detection between WHO standard Rapid Point of care test and DBS (HemaSpot-spotted samples) using the same samples from 921 Cambodian residents, the rate of agreement for the detection of HBsAg qualitatively between Rapid test and HemaSpot is very high (Supplementary Table S1a). Consistent with this perceived usefulness, our analysis yielded HBsAg, HBcAb and anti-HCV sensitivity ratios for Whatman903 and HemaSpot samples from which the measured values were the same as the estimated sensitivity ratios of serum when it was theoretically estimated by flowchart.

**Figure 5:**
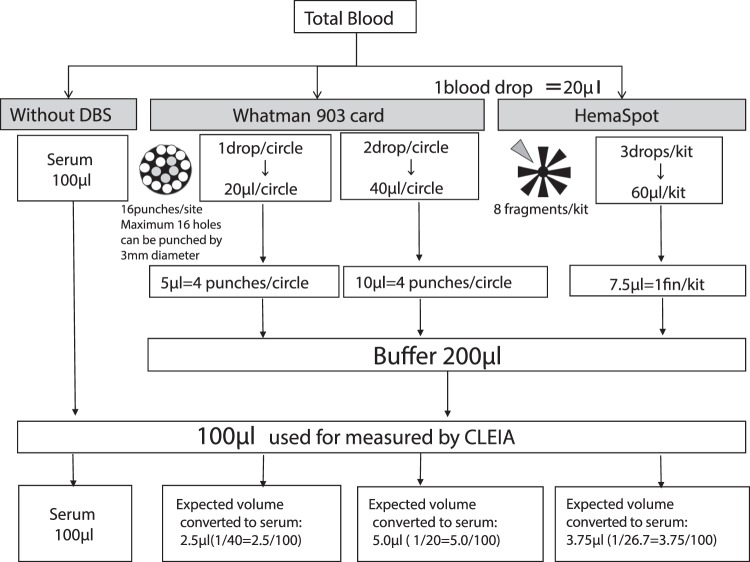
Flow chart of the conversion of Whatman903 and HemaSpotTM blood samples to serum equivalent values. The flow chart describes the conversion of values for each eluate from Whatman903 and HemaSpot samples to values equivalent to serum samples. The arrow indicates the brief procedure by which the DBS filter papers are first eluted and then diluted in an appropriate buffer solution.

Interestingly, HBsAg sensitivity ratios obtained from Whatman903 (two drops/circle) and HemaSpot (three drops/kit) samples tended to be 40–50-fold higher than those of sera at high concentrations of HBsAg. It may be probably due to application of whole blood containing high concentration which is exceeded over the maximum absorbable amount sufficiently by the filter paper used for DBS. Although our flowchart (Fig. 5) led us to expect that the measured values of hepatitis virus seromarkers from Whatman903 samples with two drops/circle would double to those measured in one drop/circle samples even this was not the observed in this study. The failure to achieve a doubled value with two drops vs. one drop might depend on the upper limit of the absorbed blood^21^ in Whatman903. This finding warrants further investigation considering its implications to using DBS in field settings where an uneven spread of blood on the filter paper may more likely occur than in laboratory settings and where the upper limit of the absorbed blood should be known and incorporated into training before collecting samples using Whatman903. Among 921 Cambodian residents, there were two samples indicated HBsAg positive of serum sample and HBsAg negative of DBS sample. The cause of the disagreement between HBsAg measured values of serum and DBS among these two samples is shown in Fig. 1. The measured amount of serum is 100 μl, but the measured amount of DBS sample is 3.75 μl in terms of serum. As HBsAg sensitivity of HemaSpot samples calculated from the ROC curve is 92.3% and specificity is 100%, both sensitivity and specificity showed more than 90%, hence HemaSpot can be used for survey in various places even in the resource limited areas as the one of DBS sampling techniques.

Regarding stability, HemaSpot yielded detection efficiencies that were 12.6–15.4% higher than those of Whatman903 at a room temperature (29 °C). Furthermore, Whatman903 appears to have a higher risk of contamination because it requires 1 hour for dry^14,22,23^ and it requires punch circles from filter paper. HemaSpot, on the other hand, requires only 10 minutes for dry, easy to transport^25^, allows for sealing and easy labeling of each kit and is designed with the absorbent paper fins which can be easily removed after opening each individually sealed kit. In brief, HemaSpot appears to be superior to Whatman903 in terms of thermos-stability and the risk of contamination.

We further observed that HBsAg and HBcAb from HemaSpot samples remained stable across different storage temperatures [−25 °C, room temperature (29 °C) and 37 °C]. On the other hands, anti-HCV from HemaSpot samples remained stable at −25 °C and room temperature (29 °C), but the sensitivity ratio of anti-HCV from Whatman903 stored at 37 °C showed high value indicating low sensitivity. Although reports on HemaSpot for humans were only literature on HIV measurement^14–17,26^, it was suggested that HBsAg, HBcAb and anti-HCV could also be measured from HemaSpot specimens through this study.

This is the first report to measure HBsAg (both qualitatively and quantitatively), HBcAb and anti-HCV from HemaSpot samples. Our analysis demonstrated that based on stability and measured hepatitis B and hepatitis C values under different storage temperatures, the analyte recovery expressed as analytical sensitivity ratio of HemaSpot was not inferior to the Whatman903 card. HemaSpot shows high rate of agreement to qualitative HBsAg rapid test and high sensitivity and specificity (sensitivity: 92.3%, specificity: 100%) in detecting HBsAg quantitatively. Not only HBsAg, the stability ans sensitivity ratio of anti-HCV using HemaSpot showed the acceptable reference value in this study. Although both Whatman903 and HemaSpot are very easy and simple methods of collecting the blood samples, HemaSpot with its compact cartridge design is more benefit in safe transportation of large quantities of samples and protects samples from contamination as well as easy labeling of samples comparing to Whatman903 which favors for the easy contamination due to lack of cover. This finding supported the reason for choosing HemaSpot as the preferable DBS sampling technique in the large sero-survey. Moreover, the HemaSpot method appears to be an easy and safe blood sampling method for children who may fear the use of syringes or 18-gauge needles for blood collection and prefer the ease, less painful and potentially high compliance with finger prick procedure. Not requiring the centrifugation of samples after blood collection as in the case of venipuncture, DBS is very useful tool in the very remote area where the human resources, technical development and the basic infrastructures such as electricity are limited.

In conclusion, this study has evaluated the usefulness of DBS sampling strategy particularly HemaSpot in term of its sensitivity, specificity and thermal stability in detecting HBV and HCV sero-markers which is the core essential step for the screening and surveillance of HBV and HCV infection. Because of its proven usefulness, we proposed that HemaSpot is acceptable as an appropriate sampling tool for large epidemiological study of HBV and HCV in all parts of the world especially in the resources limited remote areas.

## Methods

### Types of HBsAg Rapid Point of care test used in this study

1. Alere Determine^TM^ HBsAg point-of-care test strip, Abbott, Chicago, USA

### Types of DBS used in this study

(2) Whatman903 card (Whatman903 protein saver card, GE Healthcare Europe, Freiburg, Germany)
(3) HemaSpot (HemaSpot-HF, Spot On Sciences, Austin, Texas)

## Samples

### Reference samples

Reference samples were artificially generated by adding 400 µL of recombinant HBsAg that was expressed and purified from a highly differentiated human liver cancer-derived huH-1 cell line^27^ (HBsAg; 5000 IU/ml; Fujirebio Inc., Tokyo, Japan), 2000 µL of an HBV core antibody (HBcAb)-positive specimen [Cut-off Index (C.O.I): 270.5, HBV DNA: 5.7 × 10^5^ copies/ml] or 2000 µL of an HCV-antibody positive specimen (C.O.I: 91.7, HCV RNA: 1.6 × 10^7^ copies/ml) to samples of HBV and HCV seronegative blood respectively.

### Patient specimens from Japan

Three HBsAg positive and three anti-HCV positive patient specimens from Japan were used as the gold standard in comparison of the sensitivity ratios of HemaSpot samples versus serum samples.

Anti-HCV positive samples from the following patients were used:

1. Case-1: 74-year-old woman with chronic hepatitis (CH) (anti-HCV: C.O.I unmeasured, HCV RNA: 6.3 LogIU/ml)
2. Case-2: 66-year-old woman with CH (anti-HCV: C.O.I 100, HCV RNA: 6.4 LogIU/ml)
3. Case-3: 70-year-old woman with CH (anti-HCV: C.O.I 84, HCV RNA: 4.3 LogIU/ml)

HBsAg positive samples from the following patients were also used:

1. Case-4: 58-year-old woman with CH (HBsAg: 838.47 IU/ml, HBcAb: C.O.I 167, HBV DNA: 4.8 LogIU/ml)
2. Case-5: 59-year-old man with CH (HBsAg: 3177.91 IU/ml, HBcAb: C.O.I 192.8, HBV DNA: 7.2 LogIUs/ml)
3. Case-6: 83-year-old man with CH (HBsAg: 934.66 IU/ml, HBcAb: C.O.I 131.6, HBV DNA <2.1 LogIU/ml)

### Samples from Cambodia sero-survey

A total of 921 samples including four hundred HBsAg negative HemaSpot samples, all of them were initially screened and confirmed by WHO standard Rapid point of care HBsAg test, were collected using DBS(HemaSpot) and venipuncture from 5–6 years-old children and their mothers from a nationally representative epidemiological survey conducted in Cambodia in 2017and were used to evaluate the rate of agreement and specificity of DBS^28^. DBS samples were shipped at −80 degrees from Cambodia to Japan by the World Courier.

Quantitative HBsAg was measured in HemaSpot samples and serum specimens from 921 Cambodian residents (where the blood samples previously screened by Rapid test). The rate of agreement of HBsAg qualitative measurement between Rapid test and HemaSpot-spotted samples were estimated by Cohen’s kappa statistic. Correlation between serum sample and DBS samples were evaluated by Pearson’s correlation coefficient. Sensitivity and specificity of HemaSpot were calculated by ROC curves. All data were analyzed by JMP12 (SAS Institute Inc., Cary, NC, USA).

## Measurement Methods

### Measurement of HBsAg, HBcAb and anti-HCV in DBS eluates and the respective serum specimens

Automated chemiluminescent immunoassay instrument, LumipulseG1200 (Fujirebio, Inc.) was used to measure HBsAg (HBsAg-HQ, Fujirebio), HBcAb (HBcAb-N, Fujirebio) and anti-HCV (Ortho-II HCV, Fujirebio, Japan) in DBS eluates and the respective serum specimens.

### Whatman903 extraction method

Two different Whatman903 samples were prepared: In 5 circles of Whatman903, 1 drop/circle (1drop = 20 µL, in total 100 µL) and 2 drops/circle (in total 200 µL). Samples were dried at room temperature (29 °C) for 1 hour prior to storage at −25 °C for 17 hours in plastic bags containing desiccant. To elute sample from the Whatman903 card, four punched disks (diameter: 3 mm) were extracted from each circle and then transferred to separate wells of a 96-well plate. Each well contained 200 µL of elution buffer [Tris-buffered saline (TBS): 50 mM Tris, 150 mM NaCl, 0.1% Proclin 300 and 0.05% Tween 20 at pH 7.2]. After stirring the plates for 1 hour, the DBS eluates were then centrifuged at 3000 revolutions per minute (rpm) for 10 minutes and the supernatants were filtered. A chemiluminescent enzyme immunoassay (CLEIA) was then used to detect hepatitis virus seromarkers in 100-µL aliquots of the supernatants (Fig. 5).

### HemaSpot extraction method

The HemaSpot comprises absorbent filter paper separated into eight fragments in a fan-shape and a desiccant; this arrangement is covered with clear rubber in which a small central hole has been placed to allow the application of blood. Following application of the whole blood through central hole, it spreads across the eight filter paper fragments of absorbent filter paper. Three drops of whole blood (in total 60 µL) were spotted onto a HemaSpot. Subsequently, the cartridge was opened, and the sample was allowed to dry at room temperature (29 °C) for 10 minutes. The cartridge was then closed, placed into a plastic bag (FTA pouch with Silicagel, described above) and stored at −25 °C for 17 hours. Then, one fragment was detached from each HemaSpot and transferred individually into a well of a 96-well plate. The subsequent elution was performed as described above for the Whatman903 card samples. Thereafter, CLEIA was used to detect hepatitis virus seromarkers in 100-µL aliquots of supernatants from HemaSpot eluates (Fig. 5).

### Estimation of serum-converted values in Whatman903 and HemaSpot samples

The serum-converted value was 2.5 µL per drop (20 µl) of whole blood spotted over one circle on the Whatman903; four punched spots were used for measurements. Similarly, the serum-converted value was 5 µL when two drops (40 µl) of whole blood were spotted over one circle of the Whatman903; again, four punched spots from the circle were used for measurements.

For HemaSpot, the serum-converted value was 3.75 µL when three drops (60 µl) of whole blood were spotted onto the filter paper; subsequently, one fin was used for measurements (Fig. 5).

### Estimation of sensitivity ratio of HBsAg, HBcAb and anti-HCV measured using Whatman903 and HemaSpot

Because HBsAg could be measured quantitatively, the sensitivity ratios for this seromarker in DBS and serum were directly compared. The sensitivity ratio of HBsAg was calculated by (measured value from each DBS)/(serum measured value). However, as HBcAb and anti-HCV were measured qualitatively and semi-quantitatively, the resultant dilution curves (n = 2) were created using diluted fractions (2-, 4-, 8-, 16-, 32- and 64-fold) of each of the reference samples. The sensitivity ratios of HBcAb and anti-HCV were then calculated using these curves. One sample was measured twice and the mean ± SD was calculated. Further, a dilution curve was prepared by power approximation based on the average value.

According to the package insert of the assay kits, the cutoff value of HBcAb is the amount of luminescence of the standard positive solution for HBcAb × 0.9 and the cutoff value of anti-HCV is the amount of luminescence of standard positive serum for HCV × 0.28. Then, each cutoff index (C.O.I) was calculated as (amount of luminescence of specimen/cutoff value).

### Stability of HBsAg, HBcAb and anti-HCV in DBS-spotted artificial samples

To determine the stability of hepatitis virus seromarkers in DBS samples, all the artificial samples prepared using Whatman903 (1 drop and 2 drops/circle) and HemaSpot (3 drops/circle) samples were stored at two different storage temperatures [at −25 °C and at room temperature (29 °C)] for 1 month (Fig. 3). The detection efficiencies for each seromarker were calculated for the samples stored at 29 °C assuming the measured values of HBsAg, HBcAb and anti-HCV from DBS samples stored at −25 °C for 1 month have 100% detection efficacy.

### Stability of HBsAg, HBcAb and anti-HCV in HemaSpot-spotted patients’ samples

Whole blood from patients were dropped onto HemaSpot and stored at three different storage temperatures [−25 °C, room temperature (29 °C) and 37 °C] for 14 days and then sensitivity ratio for each hepatitis virus seromarker was directly compared. We compared the sensitivity ratio calculated based on the theoretical value [(serum measured value)/(estimated sensitivity ratio of DBS)] and measured value (Fig. 4). Kruskal-Wallis test was compare among sensitivity ratios of each temperature.

### Ethical consideration

Written informed consent was obtained from all participants prior to sample collection. For participants under the age of 18 years, written informed consent was obtained from a parent and/or legal guardian for study participation before samples was collected. Informed assent was obtained from all children. This study was conducted with the approval by the Ethics Committee for Epidemiological Research of Hiroshima University (E-440, E-573) and the Cambodia National Ethics Committee for Human Research (392NECHR). All methods were performed in accordance with the relevant guidelines and regulations.

## Supplementary information

Supplementary information

## Data Availability

All data generated or analyzed in this study are included in this published article and its Supplementary Information Files.
