# Supplementary material for: Evaluation of the efficiency of dried blood spot-based measurement of hepatitis B and hepatitis C virus seromarkers
Source: Sci Rep. 2020 Mar 2;10:3857. doi: 10.1038/s41598-020-60703-1 (PMC7052143; doi:10.1038/s41598-020-60703-1)
Supplement: Supplementary file 1 — Supplementary information [file 41598_2020_60703_MOESM1_ESM.doc]

**Evaluation of the efficiency of dried blood spot-based measurement of hepatitis B and hepatitis C virus seromarkers**

**Authors:** Chikako Yamamoto^1^, Shintaro Nagashima ^1^, Mitsuo Isomura², KoKo¹, Channarena Chuon¹, Tomoyuki Akita¹, Keiko Katayama¹, Joseph Woodring³^#^, Md. Shafiqul Hossain⁴, Kazuaki Takahashi¹, Junko Tanaka¹*

^1^Department of Epidemiology, Infectious Disease Control and Prevention, Graduate School of Biomedical and Health Science, Hiroshima University, Hiroshima, Japan

²Department of Clinical Evaluation, Fujirebio Inc., Tokyo, Japan

³ Expanded Program on Immunization Unit, Division of Communicable Diseases, World Health Organization Regional Office for the Western Pacific Country Office, Manila, Philippines

⁴Expanded Program on Immunization, World Health Organization Country Office, Phnom Penh, Cambodia

***Corresponding author**: Professor Junko Tanaka: Ph.D

Department of Epidemiology, Infectious Disease Control and Prevention, Graduate School of Biomedical and Health Sciences, Hiroshima University, 1-2-3 Kasumi, Minami-ku, Hiroshima 734-8551, Japan, Tel: +82-81-257-5160, Fax: +81-82-257-5164

E-mail: [jun-tanaka@hiroshima-u.ac.jp](mailto:jun-tanaka@hiroshima-u.ac.jp)

^#^Present Address of Joseph Woodring, Behavioral and Clinical Research Section, HIV/STD Research Program, Thailand MOPH - U.S. CDC Collaboration (TUC)

**Supplementary Table Legends**

**Supplementary Table S1a:** The table shows the qualitative measurement of HBsAg among 921 Cambodian general populations.

**Supplementary file**

Supplementary file S1a: HBsAg from HemaSpot samples and WHO standard Rapid point-of-care test in 921 Cambodian general populations

|  |  | **DBS (HemaSpot)** | |
| --- | --- | --- | --- |
|  |  | **(+)** | **(-)** |
| **HBsAg**  **Rapid Test** | **(+)** | **23** | **0** |
|  | **(-)** | **1** | **897** |

Cohen’s kappa statistic: K coefficient= 0.978 (95%CI: 0.94-1.0%).

**Supplementary Table S1b:** The table shows the quantitative measurement value distribution of HBsAg among 921 Cambodian general populations.

**Supplementary file**

Supplementary file S1b: HBsAg from HemaSpot samples and serum specimens in 921 Cambodian general populations

**Supplementary Table S1b**

|  | **HBsAg (C.O.I)** | | | | | | | | | | **Total** | |
| --- | --- | --- | --- | --- | --- | --- | --- | --- | --- | --- | --- | --- |
|  | **<10^-1^ (undetectable)** | | **10^-1^ – 10^0^** | | **10^0^ – 10^1^** | | **10^1^ – 10^2^** | | **>10^2^** | |  |  |
|  | **N** | **(%)** | **N** | **(%)** | **N** | **(%)** | **N** | **(%)** | **N** | **(%)** | **N** | **(%)** |
| **Serum** | 878 | 95.3% | 17 | 1.8% | 2 | 0.2% | 0 | 0.0% | 24 | 2.6% | 921 | 100.0% |
| **HemaSpot** | 884 | 96.0% | 13 | 1.4% | 1 | 0.1% | 1 | 0.1% | 22 | 2.4% | 921 | 100.0% |
